# Supplementary material for: Analyzing kinetic signaling data for G-protein-coupled receptors
Source: Sci Rep. 2020 Jul 23;10:12263. doi: 10.1038/s41598-020-67844-3 (PMC7378232; doi:10.1038/s41598-020-67844-3)
Supplement: Supplementary file 1 — Supplementary information. [file 41598_2020_67844_MOESM1_ESM.pdf]

Supplementary Information

Analyzing kinetic signaling data for G-protein-coupled receptors

Sam R.J. Hoare[1], Paul H. Tewson[2], Anne Marie Quinn[2] and Thomas E. Hughes[2]
and Lloyd J. Bridge [3]

[1] Pharmechanics, LLC, 14 Sunnyside Drive South, Owego NY 13827, USA

[2] Montana Molecular, 366 Gallatin Park Dr. Suite A, Bozeman, MT 59715 USA

[3] Department of Engineering Design and Mathematics, University of the West of England, Frenchay Campus, Bristol BS16 1QY, United Kingdom

List of Supporting Material files

Curve fit results

Curve fit results details for the analyses in the figures

Kinetic mechanism model simulations

Excel 2016 file

Time course data simulator for the mechanistic kinetic models

Signaling kinetic model-free equations

Prism 8 file

Template containing model-free equations

Signaling kinetic mechanism equations

Prism 8 file

Template containing kinetic mechanism model equations

Supplementary Figures

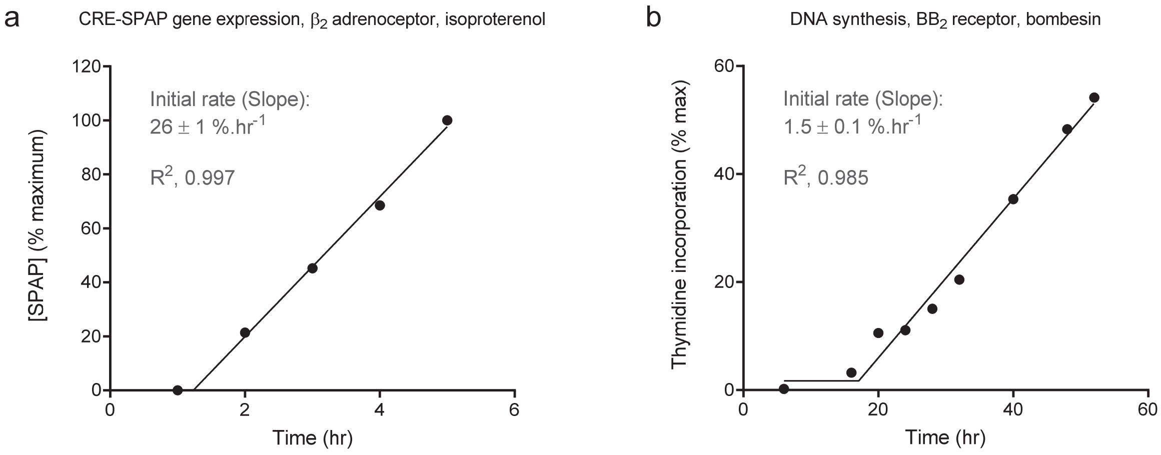

Supplemental Figure S1. Additional linear time course profiles of GPCR signaling. (a) Gene expression induction (CRE-SPAP production) via the β_2 adrenoceptor stimulated by 10 μM isoproterenol (data from Table 2 of [1]). (b) DNA synthesis in Swiss 3T3 cells stimulated by 10 nM bombesin via the BB_2 receptor (data from Fig. 2 of [2]). Data were fit to equation (5), which incorporates a delay between application of ligand (at t = 0) and initiation of response. The Slope value is the fitted value ± the fit SEM [3,4].

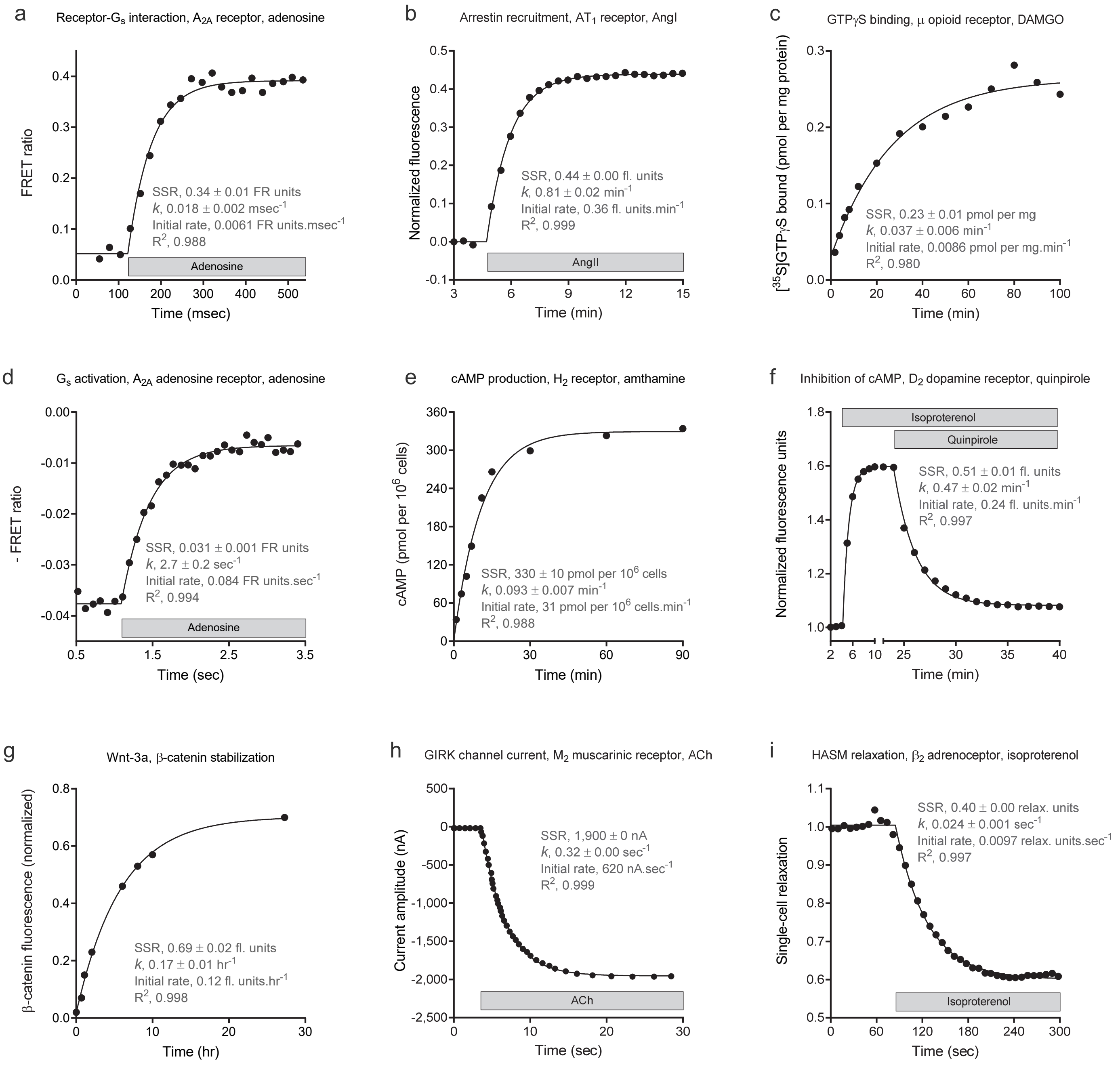

Supplemental Figure S2. Additional association exponential time course profiles of GPCR signaling. (a) Gs interaction with the A_{2A} adenosine receptor stimulated by 1 mM adenosine, measured by FRET (data from Fig. 1c of [5], fit to equation (6)). (b) Arrestin recruitment to the AT_1 angiotensin receptor stimulated by 32 μM AngII (data from Fig. 4a of [6], fit to equation (6)). (c) DAMGO-stimulated [^{35}S]GTPγS binding via the μ opioid receptor in C6 cell membranes (1 μM DAMGO, data from Fig. 1 of [7], fit to equation (2)). (d) G_s activation by the A_{2A} receptor stimulated with 100 μM adenosine, measured by FRET (data from Fig. 2c of [5], note the inverse of the FRET ratio is used). (e) cAMP production via the H_2 histamine receptor in U-937 cells stimulated by 10 μM amthamine (data from Fig. 4, D5 cell line, of [8], fit to equation (6), 1 mM isobutylmethyxanthine present). (f) Inhibition of cAMP production via the D2 dopamine receptor in HEK293T cells activated by 100 nM quinpirole. cAMP production was first stimulated by 1 nM isoproterenol via the endogenously-expressed β_2 adrenoceptor. After the plateau had been reached, quinpirole was applied and inhibition recorded. [Data are from Fig. 4a of [9], fit to equation (9) (quinpirole plus isoproterenol phase) and equation (6) (isoproterenol alone phase). Note the y values are the inverse of those in the published figure. (g) β-catenin stabilization in L cell fibroblasts stimulated by 400 ng/ml Wnt-3a (data from Fig. 4d of [10], fit to equation (2)). (h) GIRK channel gating by 1 μM acetylcholine via the M_2 muscarinic acetylcholine receptor (data from Fig. 1a of [11], fit to equation (6)). (i) Relaxation of human airway smooth muscle cells by 10 μM isoproterenol via the β_2 adrenoceptor (data from Fig. 1a of [12], fit to equation (6)). The fitted values SSR (steady-state response) and k (the rate constant) are the fitted value ± the fit SEM [3,4]. The initial rate was calculated as the SSR multiplied by k. The grey bar indicates the time interval of agonist application.

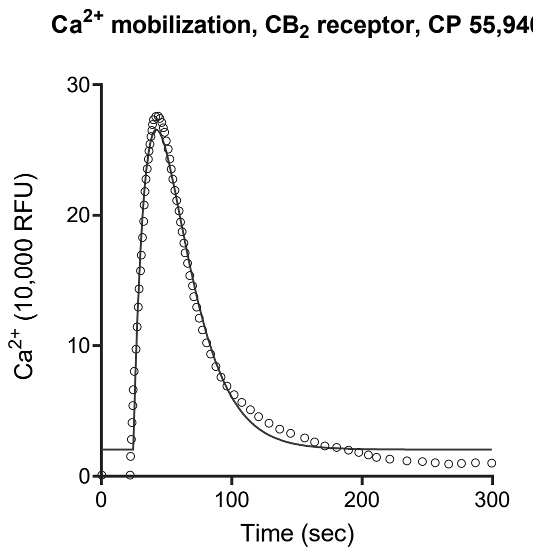

Supplemental Figure S3. Example of rise-and-fall time course data that do not fit to the model-free equations used in this study: Ca^{2+} mobilization via the $5HT_7$ receptor stimulated by 10 μM CP 55,940. The curve is the fit to the rise-and-fall to baseline equation (equation (7)). Data are from Fig. 4a of [13].

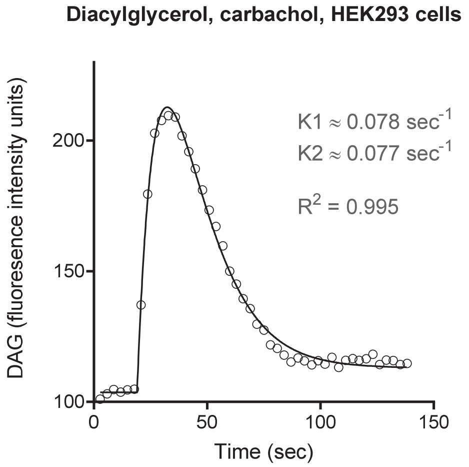

Supplemental Figure S4. Example of rise-and-fall time course profile in which the two rate constant values are almost equal and convergence was not reached: Diacylglycerol production stimulated by carbachol in HEK293 cells (data from Fig. 2A (upper panel) of [14], fit to equation (8)). For fit details, see “Curve fit results” Excel file, “Rise-and-fall to steady-state” worksheet, in Supporting Material.

Supplementary Tables

Supplementary Table S1. Summary of mechanism for straight line time course profile.

Mechanism	Scheme	Equation for saturating $[A]$
No regulation [A]	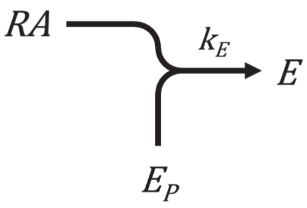	$E_{t,[A]\to\infty} = k_\tau t$

A, from ref. [15]

Supplementary Table S2. Summary of mechanisms for association exponential time course profile.

Mechanism	Scheme	Equation for saturating $[A]$	Model-free rate constant equivalence [A]
Receptor desensitization [B]	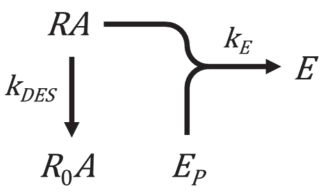	$E_{t,[A]\to\infty} = \frac{k_\tau}{k_{DES}}(1 - e^{-k_{DES}t})$	$k = k_{DES}$
Response degradation [C]	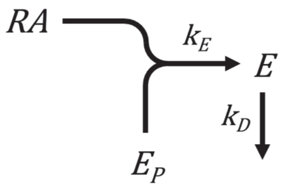	$E_{t,[A]\to\infty} = \frac{k_\tau}{k_D}(1 - e^{-k_D t})$	$k = k_D$
Response recycling [C]	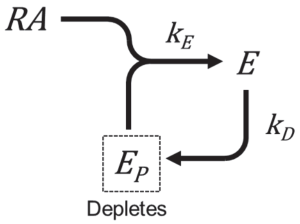	$E_{t,[A]\to\infty} = \frac{k_\tau}{k_{DEP} + k_D}\left(1 - e^{-(k_{DEP}+k_D)t}\right)$	$k = k_{DEP} + k_D$

A, rate constant k refers to the rate constant of the model-free association exponential equation, equation (2).
B, From Appendix 2.1, equation (11).
C, From ref. [15], k_{DEP} introduced in this study in place of $k_\tau/E_{P(TOT)}$ of original formulation in ref. [15]

Supplemental Table S3. Summary of mechanisms for rise-and-fall to baseline time course profiles.

Mechanism	Scheme	Equation for saturating $[A]$	Model-free rate constant equivalence [A]
Receptor desensitization and response degradation [B]	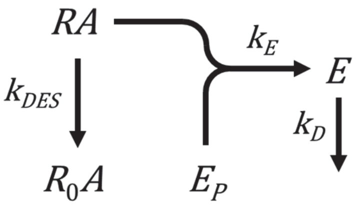	$E_{t,[A]\to\infty} = \frac{k_\tau}{k_{DES} - k_D}\left(e^{-k_D t} - e^{-k_{DES} t}\right)$	$k_1 = k_D$<br>$k_2 = k_{DES}$
Precursor depletion and response degradation [C]	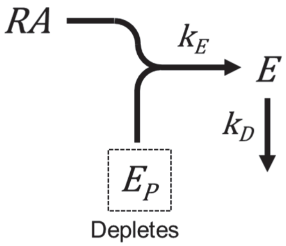	$E_{t,[A]\to\infty} = \frac{k_\tau}{k_{DEP} - k_D}\left(e^{-k_D t} - e^{-k_{DEP} t}\right)$	$k_1 = k_{DEP}$<br>$k_2 = k_D$

A, rate constants k_1 and k_2 refer to the rate constants of the model-free rise-and-fall to baseline equation, equation (3). Note k_1 was constrained to be larger than k_2 (see Methods, "Fitting Procedures") and it was assumed k_D was greater than k_{DES}, and k_{DEP} greater than k_D.

B, from Appendix 2.2, equation (12).

C, from ref. [15], k_{DEP} introduced in this study in place of $k_\tau/E_{P(TOT)}$ of original formulation in ref. [15].

Supplemental Table S4. Summary of mechanisms for rise-and-fall to steady-state time course profiles.

Mechanism	Scheme	Equation for saturating $[A]$	Model-free rate constant equivalence [A]
Receptor desensitization and resensitization with response degradation [B]	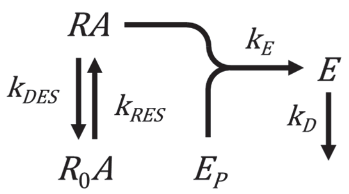	$E_{t,[A]\to\infty}=\frac{k_\tau k_{RES}}{k_D k_{DR}}\times\left[1-\frac{k_{DR}}{k_{DR}-k_D}e^{-k_D t}+\frac{k_D}{k_{DR}-k_D}e^{-k_{DR}t}\right]+\frac{k_\tau}{k_{DR}-k_D}\left(e^{-k_D t}-e^{-k_{DR}t}\right)$	$k_1=k_D$ $k_2=k_{DES}+k_{RES}$
Receptor desensitization, desensitized receptor signals, with response degradation [C]	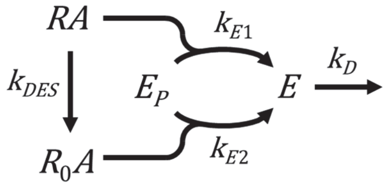	$E_{t,[A]\to\infty}=\frac{k_{\tau 2}}{k_D}\left(1-e^{-k_D t}\right)+\frac{k_{\tau 1}-k_{\tau 2}}{k_{DES}-k_D}\left(e^{-k_D t}-e^{-k_{DES}t}\right)$	$k_1=k_D$ $k_2=k_{DES}$
Response degradation to steady-state with precursor depletion [D]	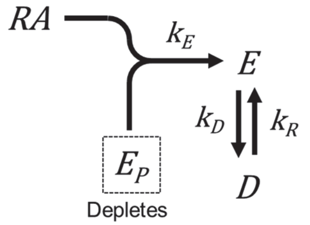	$E_{t,[A]\to\infty}=\frac{k_\tau k_R}{k_{D(obs)}k_{DEP}}\times\left[1-\frac{k_{DEP}}{k_{DEP}-k_{D(obs)}}e^{-k_{D(obs)}t}+\frac{k_{D(obs)}}{k_{DEP}-k_{D(obs)}}e^{-k_{DEP}t}\right]+\frac{k_\tau}{k_{DEP}-k_{D(obs)}}\left(e^{-k_{D(obs)}t}-e^{-k_{DEP}t}\right)$	$k_1=k_{DEP}$ $k_2=k_R+k_D$

A, rate constants k_1 and k_2 refer to the rate constants of the model-free rise-and-fall to steady-state equation, equation (4). Note k_1 was constrained to be larger than k_2 (see Methods, "Fitting Procedures") and it was assumed k_D was greater than $k_{DES}+k_{RES}$; k_D was greater than k_{DES}; and k_{DEP} greater than k_R+k_D.
B, from Appendix 2.3.1, equation (13). Note $k_{DR}=k_{DES}+k_{RES}$.
C, from Appendix 2.3.2, equation (14).
D, from Appendix 2.3.3, equation (16). Note $k_{D(obs)}=k_R+k_D$.

Supplementary Table S5. Definition of model parameters

Term	Description	Units
k_τ	Transduction rate constant - $E_P[R]_{TOT}k_E$	Response units.t^{-1}
ρ_A	Fractional receptor occupancy by agonist - $[A]^n/({K_A}^n + [A]^n)$	Unitless
$[A]$	Agonist concentration	Molar
K_A	Agonist equilibrium dissociation constant	Molar
n	Agonist binding slope factor	Unitless
$[R]_{TOT}$	Total receptor concentration	Receptor units
$[R_a]_{TOT}$	Total non-desensitized receptor concentration	Receptor units
$[R_0]_{TOT}$	Total desensitized receptor concentration	Receptor units
E	Response	Response units
E_P	Response precursor	Response units
$E_{P(TOT)}$	Total response precursor	Response units
D	Response degradation product	Response units
k_E	Response generation rate constant	Receptor units^{-1}.t^{-1}
k_{DES}	Receptor desensitization rate constant	t^{-1}
k_{RES}	Receptor resensitization rate constant	t^{-1}
k_D	Response degradation rate constant	t^{-1}
k_R	Response reformation rate constant	t^{-1}
$k_{D(obs)}$	Observed response degradation rate constant - $k_R + k_D$	t^{-1}
k_{DEP}	Response precursor depletion rate constant - $[R]_{TOT}k_E$	t^{-1}
k_{DR}	$k_{DES} + k_{RES}$	t^{-1}
$k_{\tau 1}$ [A]	Transduction rate constant of non-desensitized receptor	Response units.t^{-1}
$k_{\tau 2}$ [A]	Transduction rate constant of desensitized receptor	Response units.t^{-1}
k_{E1} [A]	Response generation rate constant of non-desensitized receptor	Receptor units^{-1}.t^{-1}
k_{E2} [A]	Response generation rate constant of desensitized receptor	Receptor units^{-1}.t^{-1}

A, Applies to receptor desensitization, desensitized receptor signals model (Appendix 2.3.2).

Model equations list

These equations are loaded into Prism 8 as user-defined equations in a template called “Signaling kinetic mechanism equations” available in the supporting files.

Note in all cases,

$$\rho_A = \frac{[A]^n}{{K_A}^n + [A]^n}$$

No regulation

$E_t = \rho_A k_\tau t$

Equation (1) of ref. [15]

No regulation, max A

$E_{t,[A]\to\infty} = k_\tau t$

Receptor desensitization, max A

$E_{t,[A]\to\infty} = \frac{k_\tau}{k_{DES}}(1 - e^{-k_{DES}t})$

Equation (11) from Appendix 2.1. Note model was formulated only for saturating $[A]$.

Response degradation

$E_t = \frac{\rho_A k_\tau}{k_D}(1 - e^{-k_D t})$

Equation (2) of ref. [15]

Response degradation, max A

$E_{t,[A]\to\infty} = \frac{k_\tau}{k_D}(1 - e^{-k_D t})$

Response recycling

$$E_t = \frac{\rho_A k_\tau}{k_{obs}}(1 - e^{-k_{obs}t})$$
$$k_{obs} = \rho_A k_{DEP} + k_D$$

Equation (3) or ref. [15], modified so that k_{DEP} replaces $k_\tau / E_{P(TOT)}$.

Response recycling, max A

$$E_{t,[A]\to\infty} = \frac{k_\tau}{k_{obs}}(1 - e^{-k_{obs}t})$$

Note $k_{obs} = k_{DEP} + k_D$ but k_{DEP} and k_D cannot be independently identified with saturating $[A]$.

Receptor desensitization & response degradation, max A

$$E_{t,[A]\to\infty} = \frac{k_\tau}{k_{DES} - k_D}\left(e^{-k_D t} - e^{-k_{DES}t}\right)$$

Equation (12) from Appendix 2.2. Note model was formulated only for saturating $[A]$.

Precursor depletion & response degradation

$$E_t = \frac{\rho_A k_\tau}{\rho_A k_{DEP} - k_D}\left(e^{-k_D t} - e^{-\rho_A k_{DEP} t}\right)$$

Equation (4) or ref. [15], modified so that k_{DEP} replaces $k_\tau / E_{P(TOT)}$.

Precursor depletion & response degradation, max A

$$E_t = \frac{k_\tau}{k_{DEP} - k_D}\left(e^{-k_D t} - e^{-k_{DEP}t}\right)$$

Note, k_{DEP} assumed to be greater than k_D, constrained to be so in fit.

Receptor desensitization & resensitization, & response degradation, max A

$$E_{t,[A]\to\infty} = \frac{k_\tau k_{RES}}{k_D k_{DR}}\left[1 - \frac{k_{DR}}{k_{DR} - k_D}e^{-k_D t} + \frac{k_D}{k_{DR} - k_D}e^{-k_{DR}t}\right] + \frac{k_\tau}{k_{DR} - k_D}(e^{-k_D t} - e^{-k_{DR}t})$$
$$k_{DR} = k_{DES} + k_{RES}$$

Equation (13) from Appendix 2.3.1. Note model was formulated only for saturating $[A]$.

Receptor desensitization, desensitized receptor signals, & response degradation, max A

$$E_{t,[A]\to\infty} = \frac{k_{\tau 2}}{k_D}(1 - e^{-k_D t}) + \frac{k_{\tau 1} - k_{\tau 2}}{k_{DES} - k_D}(e^{-k_D t} - e^{-k_{DES} t})$$

Equation (14) from Appendix 2.3.2. Note model was formulated only for saturating $[A]$.

Precursor depletion & response degradation to steady-state

$$E_t = \frac{k_\tau k_R}{k_{D(obs)} k_{DEP}}\left[1 - \frac{\rho_A k_{DEP}}{\rho_A k_{DEP} - k_{D(obs)}} e^{-k_{D(obs)} t} + \frac{k_{D(obs)}}{\rho_A k_{DEP} - k_{D(obs)}} e^{-\rho_A k_{DEP} t}\right] + \frac{\rho_A k_\tau}{\rho_A k_{DEP} - k_{D(obs)}}\left(e^{-k_{D(obs)} t} - e^{-\rho_A k_{DEP} t}\right)$$

$$k_{D(obs)} = k_R + k_D$$

Equation (15) from Appendix 2.3.3.

Precursor depletion & response degradation to steady-state, max A

$$E_t = \frac{k_\tau k_R}{k_{D(obs)} k_{DEP}}\left[1 - \frac{k_{DEP}}{k_{DEP} - k_{D(obs)}} e^{-k_{D(obs)} t} + \frac{k_{D(obs)}}{k_{DEP} - k_{D(obs)}} e^{-k_{DEP} t}\right] + \frac{k_\tau}{k_{DEP} - k_{D(obs)}}\left(e^{-k_{D(obs)} t} - e^{-k_{DEP} t}\right)$$

$$k_{D(obs)} = k_R + k_D$$

Equation (16) from Appendix 2.3.3.

References

1 Baker, J. G., Hall, I. P. & Hill, S. J. Temporal characteristics of cAMP response element-mediated gene transcription: requirement for sustained cAMP production. *Mol Pharmacol* **65**, 986-998, doi:10.1124/mol.65.4.986 (2004).

2 Withers, D. J. *et al.* Rapamycin dissociates p70(S6K) activation from DNA synthesis stimulated by bombesin and insulin in Swiss 3T3 cells. *J Biol Chem* **272**, 2509-2514, doi:10.1074/jbc.272.4.2509 (1997).

3 Motulsky, H. J. *How standard errors are computed.* *https://www.graphpad.com/guides/prism/8/curve-fitting/reg_how_standard_errors_are_comput.htm?q=standard+error*, (2019).

4 Motulsky, H. J. *Standarad error of parameters.* *https://www.graphpad.com/guides/prism/8/curve-fitting/reg_standarad_error_of_parameters.htm?q=standard+error*, (2019).

5 Hein, P. *et al.* Gs activation is time-limiting in initiating receptor-mediated signaling. *J Biol Chem* **281**, 33345-33351, doi:10.1074/jbc.M606713200 (2006).

6 Hoare, S. R. J., Tewson, P., Quinn, A. M. & Hughes, T. A kinetic method for measuring agonist efficacy and ligand bias using high resolution biosensors and a kinetic data analysis framework. *BioRxiv* **https://doi.org/10.1101/772293** (2019).

7 Traynor, J. R., Clark, M. J. & Remmers, A. E. Relationship between rate and extent of G protein activation: comparison between full and partial opioid agonists. *J Pharmacol Exp Ther* **300**, 157-161 (2002).

8 Fernandez, N. *et al.* Reduction of G protein-coupled receptor kinase 2 expression in U-937 cells attenuates H2 histamine receptor desensitization and induces cell maturation. *Mol Pharmacol* **62**, 1506-1514, doi:10.1124/mol.62.6.1506 (2002).

9 Tewson, P. *et al.* Assay for Detecting Galphai-Mediated Decreases in cAMP in Living Cells. *SLAS Discov* **23**, 898-906, doi:10.1177/2472555218786238 (2018).

10 Hannoush, R. N. Kinetics of Wnt-driven beta-catenin stabilization revealed by quantitative and temporal imaging. *PLoS One* **3**, e3498, doi:10.1371/journal.pone.0003498 (2008).

11 Zhang, Q., Pacheco, M. A. & Doupnik, C. A. Gating properties of GIRK channels activated by Galpha(o)- and Galpha(i)-coupled muscarinic m2 receptors in Xenopus oocytes: the role of receptor precoupling in RGS modulation. *J Physiol* **545**, 355-373, doi:10.1113/jphysiol.2002.032151 (2002).

12 Ojiaku, C. A. *et al.* Transforming Growth Factor-beta1 Decreases beta2-Agonist-induced Relaxation in Human Airway Smooth Muscle. *Am J Respir Cell Mol Biol* **61**, 209-218, doi:10.1165/rcmb.2018-0301OC (2019).

13 Malysz, J. *et al.* Characterization of human cannabinoid CB2 receptor coupled to chimeric Galpha(qi5) and Galpha(qo5) proteins. *Eur J Pharmacol* **603**, 12-21, doi:10.1016/j.ejphar.2008.11.047 (2009).

14 Tewson, P. H., Quinn, A. M. & Hughes, T. E. A multiplexed fluorescent assay for independent second-messenger systems: decoding GPCR activation in living cells. *J Biomol Screen* **18**, 797-806, doi:10.1177/1087057113485427 (2013).

15 Hoare, S. R. J., Pierre, N., Moya, A. G. & Larson, B. Kinetic operational models of agonism for G-protein-coupled receptors. *J Theor Biol* **446**, 168-204, doi:10.1016/j.jtbi.2018.02.014 (2018).
