# Supplementary material for: Analyzing kinetic signaling data for G-protein-coupled receptors
Source: Sci Rep. 2020 Jul 23;10:12263. doi: 10.1038/s41598-020-67844-3 (PMC7378232; doi:10.1038/s41598-020-67844-3)
Supplement: Supplementary file 2 — Appendix. [file 41598_2020_67844_MOESM2_ESM.docx]

# Appendix 1. Initial rate derivation

The initial rate of a biological process is the rate before it becomes limited by rate-limiting mechanisms. It is the rate of the process as time approaches zero. The parameters of the time course equation defining the initial rate can be identified by taking the limit of the equation as time approaches zero.

## 1.1. Initial rate of model-free equations

The limit of the model-free equations (excluding Baseline) as time approaches zero is as follows:

Straight line, from equation (1):

$$y_{t\to0}=Slope.t$$

Association exponential, from equation (2):

$$y_{t\to0}=\mathrm{SSR}.k.t$$

Rise-and-fall to baseline, from equation (3):

$$y_{t\to0}=C.t$$

Rise-and-fall to steady-state, from equation (4):

$$y_{t\to0}=SSR.\left( Dk_{1}-\left( D-1 \right)k_{2} \right).t$$

These are all straight line equations. The initial rate ($\mathrm{IR}$) is the gradient of the line:

Straight line:

$$\mathrm{IR}=Slope$$

Association exponential:

$$IR=SSR\times k$$

Rise-and-fall to baseline:

$$IR=C$$

Rise-and-fall to steady-state:

$$IR=SSR\times\left( Dk_{1}-\left( D-1 \right)k_{2} \right)$$

## 1.2. Initial rate of kinetic model equations

Efficacy of the receptor-ligand complex is determined using a saturating concentration of agonist. The equations for the kinetic mechanistic models at saturating $[A]$ are given in Supplementary Tables S1-S4. Taking the limit of these equations as time approaches zero, in all cases the equations reduce to the following:

$$E_{t\to0,\left[ A \right]\to\infty}=k_{\tau}$$

(Note in the case of equation (14) the limit is $k_{\tau1}$, the $k_{\tau}$ value of the non-desensitized receptor.) This finding indicates that the initial rate at saturating $[A]$ from the model-free analysis is equivalent to $k_{\tau}$.

# Appendix 2. More complex models

Kinetic mechanistic models were developed here that incorporate receptor desensitization. These models employ the same formulation used in the kinetic mechanistic model ^33^. Agonist-bound receptor ($RA$) converts response precursor ($E_{P}$) to the response ($E$) governed by the response generation rate constant $k_{E}$. Here receptor desensitization is incorporated as a decrease of the receptor concentration that can generate the response; active receptor ($RA$) is converted to inactive receptor ($R_{0}A$) governed by the desensitization rate constant $k_{DES}$. Four models are considered – desensitization alone (Appendix 2.1); desensitization with response decay (Appendix 2.2); desensitization with resensitization and response decay (Appendix 2.3.1); and desensitization in which the desensitized receptor signals at a different rate (Appendix 2.3.2). Note the models assume the level of receptor-ligand complex does not change over time. This scenario likely applies to maximally-stimulating concentrations of ligand (used to quantify efficacy), and for all concentrations of lower potency ligands, as described in the Discussion. The model framework allows for extension to incorporate receptor-ligand binding kinetics as necessary ^33^.

## 2.1. Desensitization alone model

The basic model is described by Scheme 1:


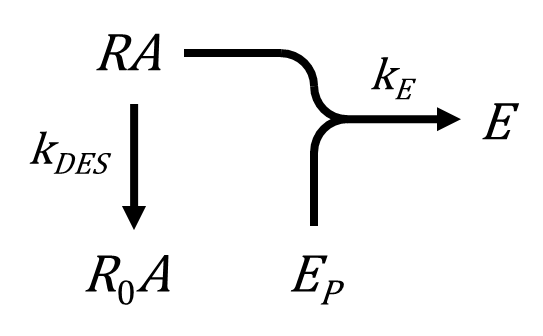


**Scheme 1**

In this particular model, the response does not decay and the response precursor concentration remains constant over time, i.e. it is not depleted by generation of the response. The model is formulated for a maximally-stimulating concentration of agonist rather than a range of concentrations of agonist. This avoids the complexity of considering differential affinity for active versus desensitized receptors. The goal is an analytical equation for $E$, termed here an *E vs t* equation, which can be obtained using the Laplace transform method as follows. The differential equation for $E$ is,

$$\frac{dE}{dt}=E_{P}[RA]k_{E}$$

Since the concentration of agonist is saturating, $\left[ RA \right]$ does not change over time and is equal to the total concentration of non-desensitized receptors, termed ${[R_{a}]}_{TOT}$. The Laplace transform is then,

$$s\bar{E}=E_{P}\bar{{[R_{a}]}_{TOT}}k_{E}$$

The differential equation for ${[R_{a}]}_{TOT}$ is,

$$\frac{d\left[ R_{a} \right]_{TOT}}{dt}=-\left[ R_{a} \right]_{TOT}k_{DES}$$

with boundary condition $\left[ R_{a} \right]_{TOT,t=0}={[R]}_{TOT}$. The Laplace transform for $[RA]$ is then equation (10),

$$\bar{\left[ R_{a} \right]_{TOT}}=\frac{{[R]}_{TOT}}{s+k_{DES}}$$

equation (10)

This is now substituted into the transform for $E$ which gives, after re-arranging,

$$\bar{E}=\frac{E_{P}{[R]}_{TOT}k_{E}}{s\left( s+k_{DES} \right)}$$

The $k_{\tau}$ term is now introduced. This is the initial rate of signal generation by the agonist-occupied receptor, the product of the total precursor concentration, total receptor concentration and the response generation rate constant ^33^, specifically:

$$k_{\tau}=E_{P}{[R]}_{TOT}k_{E}$$

Substituting gives,

$$\bar{E}=\frac{k_{\tau}}{s\left( s+k_{DES} \right)}$$

The analytic equation, equation (11) is now obtained by taking the inverse Laplace transform:

$$E_{t}=\frac{k_{\tau}}{k_{DES}}\left( 1-e^{-k_{DES}t} \right)$$

equation (11)

## 2.2. Receptor desensitization with response degradation model

This model combines receptor desensitization with a second regulation of signaling mechanism, degradation of the response (for example, breakdown of second messenger molecules). (The response degradation model was described in the original kinetic mechanistic model ^33^.) The mechanism is described by Scheme 2:


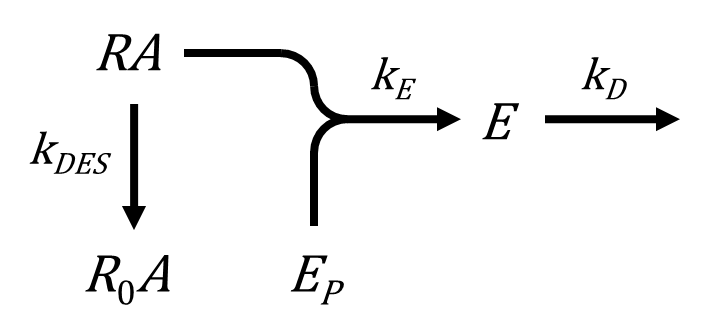


**Scheme 2**

The *E vs t* equation is obtained, for a saturating agonist concentration, as follows. The differential equation and Laplace transform for $E$ are,

$$\frac{dE}{dt}=E_{P}\left[ R_{a} \right]_{TOT}k_{E}-Ek_{D}$$

$$s\bar{E}=E_{P}\bar{\left[ R_{a} \right]_{TOT}}k_{E}-\bar{E}k_{D}$$

The derivation proceeds by taking the Laplace transform for $\left[ R_{a} \right]_{TOT}$ (equation (10), Appendix 2.1) and substituting it into the transform for $E$, giving, after solving for $\bar{E}$,

$$\bar{E}=\frac{k_{\tau}}{\left( s+k_{D} \right)\left( s+k_{DES} \right)}$$

The *E vs t* equation is now obtained by taking the inverse Laplace transform, giving equation (12):

$$E_{t}=\frac{k_{\tau}}{k_{DES}-k_{D}}\left( e^{-k_{D}t}-e^{-k_{DES}t} \right)$$

equation (12)

## 2.3. Rise-and-fall to steady-state models

More complex regulation mechanisms have been described for GPCRs beyond the canonical receptor desensensitization and response degradation mechanisms. Three of these models are described and formulated here. All three reduce to a common general equation, the rise-and-fall to steady-state equation (equation (4)).

### 2.3.1. Receptor desensitization and resensitization with response degradation

In this model, the receptor resensitizes after desensitizing. This is represented as a return to the active receptor state, $RA$, from the desensitized state $R_{0}A$. This process proceeds at a rate defined by $k_{RES}$, the resensitization rate constant. The mechanism, including response degradation, is an extension of the model in Appendix 2.2, and is represented by Scheme 3 below:


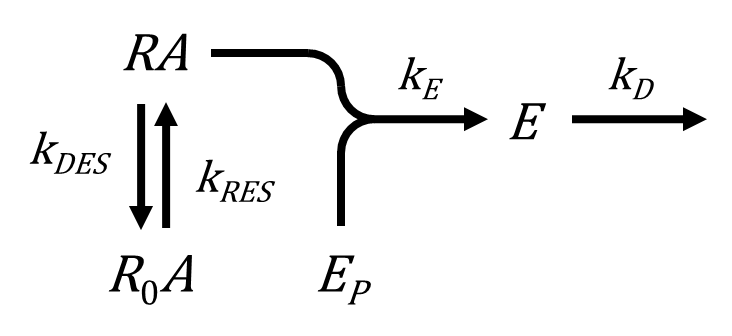


**Scheme 3**

Here the model is formulated for a saturating concentration of agonist. This simplifies the model by avoiding consideration of the fate of desensitized receptors that become unbound by the agonist. The *E vs t* equation can be obtained using Laplace transforms as follows. First, the differential equation and Laplace transform for $E$ are,

$$\frac{dE}{dt}=E_{P}{[R_{a}]}_{TOT}k_{E}-Ek_{D}$$

$$s\bar{E}=E_{P}\bar{{[R_{a}]}_{TOT}}k_{E}-\bar{E}k_{D}$$

Note since we are dealing with a saturating concentration of agonist, $[RA]$ is equal to ${[R_{a}]}_{TOT}$, the total concentration of non-desensitized receptors. The derivation proceeds by taking the Laplace transform for ${[R_{a}]}_{TOT}$ and substituting it into the transform for $E$. ${[R_{a}]}_{TOT}$ changes over time due to $RA$ desensitization to $R_{0}A$ and resensitization of $R_{0}A$ back to $RA$. The resulting differential equation for ${[R_{a}]}_{TOT}$ is, for a saturating concentration of agonist,

$$\frac{d{[R_{a}]}_{TOT}}{dt}={[R_{0}]}_{TOT}k_{RES}-{[R_{a}]}_{TOT}k_{DES}$$

The ${[R_{0}]}_{TOT}$ term, the total concentration of desensitized receptors, can be replaced using the conservation of mass equation for the receptor (${[R]}_{TOT}={[R_{a}]}_{TOT}+{[R_{0}]}_{TOT}$):

$$\frac{d{[R_{a}]}_{TOT}}{dt}={[R]}_{TOT}k_{RES}-{[R_{a}]}_{TOT}k_{DR}$$

where $k_{DR}=k_{DES}+k_{RES}$. The Laplace transform is,

$$s\bar{{[R_{a}]}_{TOT}}={[R]}_{TOT}+\frac{{[R]}_{TOT}k_{RES}}{s}-\bar{{[R_{a}]}_{TOT}}k_{DR}$$

Note the transform includes ${[R_{a}]}_{TOT}$ at the initiation of the experiment and that this equals to the total concentration of receptors (${[R]}_{TOT})$ because desensitization has yet to take place. Solving for $\bar{{[R_{a}]}_{TOT}}$ gives,

$$\bar{{[R_{a}]}_{TOT}}=\frac{{[R]}_{TOT}k_{RES}}{s\left( s+k_{DR} \right)}+\frac{{[R]}_{TOT}}{s+k_{DR}}$$

Substituting into the transform for $E$ and solving for $\bar{E}$ gives,

$$\bar{E}=\frac{k_{\tau}k_{RES}}{s\left( s+k_{D} \right)\left( s+k_{DR} \right)}+\frac{k_{\tau}}{\left( s+k_{D} \right)\left( s+k_{DR} \right)}$$

Taking the inverse Laplace transform gives the *E vs t* equation, equation (13):

$$E_{t,[A]\to\infty}=\frac{k_{\tau}k_{RES}}{k_{D}k_{DR}}\left[ 1-\frac{k_{DR}}{k_{DR}-k_{D}}e^{-k_{D}t}+\frac{k_{D}}{k_{DR}-k_{D}}e^{-k_{DR}t} \right]+\frac{k_{\tau}}{k_{DR}-k_{D}}\left( e^{-k_{D}t}-e^{-k_{DR}t} \right)$$

equation (13)

The equation can be rearranged to the general form:

$$E_{t}=SSR\times\left( 1-De^{-k_{1}t}+\left( D-1 \right)e^{-k_{2}t} \right)$$

where $\mathrm{SSR}$ is the steady-state response, i.e. response as $t\to\infty$. This rearrangement involves the intermediate step:

$$E_{t,[A]\to\infty}=\frac{k_{\tau}k_{RES}}{k_{D}k_{DR}}\left( 1-\frac{k_{DR}\left( k_{RES}-k_{D} \right)}{k_{RES}\left( k_{DR}-k_{D} \right)}e^{-k_{D}t}+\frac{k_{D}\left( k_{RES}-k_{DR} \right)}{k_{RES}\left( k_{DR}-k_{D} \right)}e^{-k_{DR}t} \right)$$

and the observation that the $e^{-k_{DR}t}$ multiplier equals the $e^{-k_{D}t}$ multiplier minus unity, i.e:

$$\frac{k_{D}\left( k_{RES}-k_{DR} \right)}{k_{RES}\left( k_{DR}-k_{D} \right)}=\frac{k_{DR}\left( k_{RES}-k_{D} \right)}{k_{RES}\left( k_{DR}-k_{D} \right)}-1$$

The parameters are defined as follows:

$$SSR=\frac{k_{\tau}k_{RES}}{k_{D}k_{DR}}$$

$$D=\frac{k_{DR}\left( k_{RES}-k_{D} \right)}{k_{RES}\left( k_{DR}-k_{D} \right)}$$

$$k_{1}=k_{D}$$

$$k_{2}=k_{DR}$$

The initial rate of the response is $k_{\tau}$, shown by taking the limit of equation (13) as time approaches zero:

$$E_{t,\left[ A \right]\to\infty,t\to0}=k_{\tau}$$

### 2.3.2. Receptor desensitization, desensitized receptor signals

Recently it has been discovered that certain receptors which become desensitized can remain active for signaling. This mechanism is represented here by assuming the desensitized receptor $R_{0}A$ can couple to $E_{P}$ to generate the response. The response generation rate constant of desensitized and non-desensitized receptor is assumed to be different (defined by $k_{E2}$ and $k_{E1}$, respectively). Here the simplest model is considered, one in which there is no receptor resesensitization, no precursor depletion, and a single rate of response degradation. It is represented by Scheme 4:


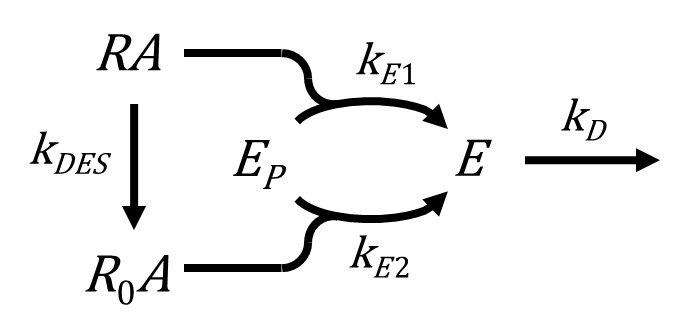


**Scheme 4**

Here the model is formulated for a maximally-stimulating concentration of agonist. The differential equation for $E$ is,

$$\frac{dE}{dt}=E_{P}\left[ R_{a} \right]_{TOT}k_{E1}+E_{P}\left[ R_{0} \right]_{TOT}k_{E2}-Ek_{D}$$

This can be simplified by employing the conservation of mass equation for the receptor, $\left[ R \right]_{TOT}=\left[ R_{a} \right]_{TOT}+\left[ R_{0} \right]_{TOT}$:

$$\frac{dE}{dt}=E_{P}\left[ R \right]_{TOT}k_{E2}+E_{P}\left[ R_{a} \right]_{TOT}\left( k_{E1}-k_{E2} \right)-Ek_{D}$$

The Laplace transform is,

$$s\bar{E}=\frac{E_{P}\left[ R \right]_{TOT}k_{E2}}{s}+E_{P}\bar{\left[ R_{a} \right]_{TOT}}\left( k_{E1}-k_{E2} \right)-\bar{E}k_{D}$$

The Laplace transform for ${[R_{a}]}_{TOT}$ is equation (10) (Appendix 2.1):

$$\bar{\left[ R_{a} \right]_{TOT}}=\frac{\left[ R \right]_{TOT}}{s+k_{DES}}$$

Substituting into the transform for $E$ gives,

$$s\bar{E}=\frac{E_{P}\left[ R \right]_{TOT}k_{E2}}{s}+\frac{E_{P}\left[ R \right]_{TOT}\left( k_{E1}-k_{E2} \right)}{s+k_{DES}}-\bar{E}k_{D}$$

$k_{\tau}$ is now introduced. There are two terms, for non-desensitized and desensitized receptors, defined respectively as,

$$k_{\tau1}=E_{P}\left[ R \right]_{TOT}k_{E1}$$

$$k_{\tau2}=E_{P}\left[ R \right]_{TOT}k_{E2}$$

Substituting and solving for $\bar{E}$ gives,

$$\bar{E}=\frac{k_{\tau2}}{s\left( s+k_{D} \right)}+\frac{k_{\tau1}-k_{\tau2}}{\left( s+k_{D} \right)\left( s+k_{DES} \right)}$$

Taking the inverse Laplace transform gives the *E vs t* equation, equation (14)

$$E_{t,[A]\to\infty}=\frac{k_{\tau2}}{k_{D}}\left( 1-e^{-k_{D}t} \right)+\frac{k_{\tau1}-k_{\tau2}}{k_{DES}-k_{D}}\left( e^{-k_{D}t}-e^{-k_{DES}t} \right)$$

equation (14)

The equation can be rearranged to the general form:

$$E_{t}=SSR\times\left( 1-De^{-k_{1}t}+\left( D-1 \right)e^{-k_{2}t} \right)$$

where $\mathrm{SSR}$ is the steady-state response, i.e. response as $t\to\infty$. This rearrangement involves the intermediate step:

$$E_{t,[A]\to\infty}=\frac{k_{\tau2}}{k_{D}}\left( 1-\frac{k_{\tau2}k_{DES}-k_{D}k_{\tau1}}{k_{\tau2}\left( k_{DES}-k_{D} \right)}e^{-k_{D}t}+\frac{k_{D}\left( k_{\tau2}-k_{\tau1} \right)}{k_{\tau2}\left( k_{DES}-k_{D} \right)}e^{-k_{DES}t} \right)$$

and the observation that the $e^{-k_{DES}t}$ multiplier equals the $e^{-k_{D}t}$ multiplier minus unity, i.e:

$$\frac{k_{D}\left( k_{\tau2}-k_{\tau1} \right)}{k_{\tau2}\left( k_{DES}-k_{D} \right)}=\frac{k_{\tau2}k_{DES}-k_{D}k_{\tau1}}{k_{\tau2}\left( k_{DES}-k_{D} \right)}-1$$

The parameters are defined as follows:

$$SSR=\frac{k_{\tau2}}{k_{D}}$$

$$D=\frac{k_{\tau2}k_{DES}-k_{D}k_{\tau1}}{k_{\tau2}\left( k_{DES}-k_{D} \right)}$$

$$k_{1}=k_{D}$$

$$k_{2}=k_{DES}$$

The initial rate of the response is $k_{\tau1}$, shown by taking the limit of equation (14) as time approaches zero:

$$E_{t,\left[ A \right]\to\infty,t\to0}=k_{\tau1}$$

### 2.3.3. Precursor depletion & response degradation to steady-state

In the calcium signaling mechanism, after the calcium rise and fall, the processes modulating cytoplasmic Ca^2+^ can reach a steady-state that results in a constant level of Ca^2+^ over time ^68,72,73,75^. The steady-state between Ca^2+^ mobilization and clearance can be accommodated within the model as an extension of the original precursor depletion and response degradation model (Model 4 in ^33^). The response degradation step becomes reversible. In other words, the response degradation product converts back to the response. This is represented by Scheme 5 below:


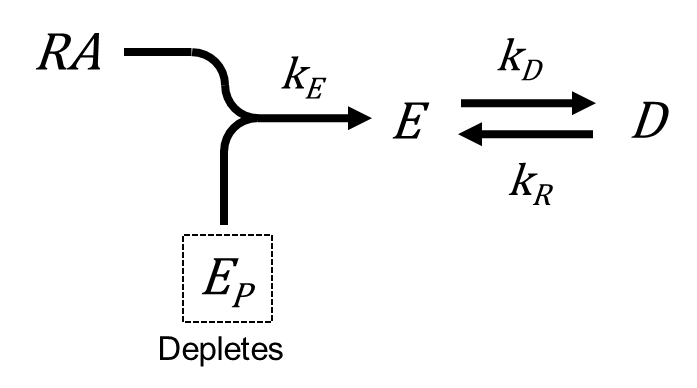


**Scheme 5**

where $D$ is the response degradation product and $k_{R}$ is the response reformation rate constant. The differential equations for $E$ is:

$$\frac{dE}{dt}=E_{P}\left[ RA \right]k_{E}+Dk_{R}-Ek_{D}$$

$\left[ RA \right]$ can be expressed as a function of the total receptor concentration:

$$\left[ RA \right]=\rho_{A}{[R]}_{TOT}$$

where $\rho_{A}$ is fractional occupancy of receptor by agonist, given by the standard equilibrium receptor-ligand occupancy function:

$$\rho_{A}=\frac{{[A]}^{n}}{{K_{A}}^{n}+{[A]}^{n}}$$

$[A]$ is agonist concentration, $K_{A}$ is the agonist-receptor equilibrium dissociation constant and $n$ the slope factor.

$E_{P}$ can be substituted using the conservation of mass equation, $E_{P\left( TOT \right)}=E_{P}-E-D$:

$$\frac{dE}{dt}=E_{P\left( TOT \right)}\rho_{A}{[R]}_{TOT}k_{E}-D\left( \rho_{A}{[R]}_{TOT}k_{E}-k_{R} \right)-E\left( \rho_{A}{[R]}_{TOT}k_{E}+k_{D} \right)$$

$D$ can now be replaced with an expression in terms of $E$ using Laplace transforms. The transform for $E$ is,

$$s\bar{E}=\frac{E_{P\left( TOT \right)}\rho_{A}{[R]}_{TOT}k_{E}}{s}-\bar{D}\left( \rho_{A}{[R]}_{TOT}k_{E}-k_{R} \right)-\bar{E}\left( \rho_{A}{[R]}_{TOT}k_{E}+k_{D} \right)$$

The differential equation and transform for $D$ is,

$$\frac{dD}{dt}=Ek_{D}-Dk_{R}$$

$$s\bar{D}=\bar{E}k_{D}-\bar{D}k_{R}$$

Solving for $\bar{D}$ and substituting into the transform for $E$ and solving for $\bar{E}$, gives:

$$\bar{E}=\frac{\rho_{A}k_{\tau}k_{R}}{s\left( s+k_{D(obs)} \right)\left( s+\rho_{A}k_{DEP} \right)}+\frac{\rho_{A}k_{\tau}}{\left( s+k_{D(obs)} \right)\left( s+\rho_{A}k_{DEP} \right)}$$

$k_{DEP}$ is response precursor depletion rate constant, defined as the product of the total receptor concentration and the response generation rate constant, i.e. $k_{DEP}={[R]}_{TOT}k_{E}$. The term $k_{D(obs)}$ is $k_{R}+k_{D}$. Taking the inverse Laplace transform gives the *E vs t* equation, equation (15):

$$E_{t}=\frac{k_{\tau}k_{R}}{k_{D(obs)}k_{DEP}}\left[ 1-\frac{\rho_{A}k_{DEP}}{\rho_{A}k_{DEP}-k_{D(obs)}}e^{-k_{D(obs)}t}+\frac{k_{D(obs)}}{\rho_{A}k_{DEP}-k_{D(obs)}}e^{-\rho_{A}k_{DEP}t} \right]+\frac{\rho_{A}k_{\tau}}{\rho_{A}k_{DEP}-k_{D(obs)}}\left( e^{-k_{D(obs)}t}-e^{-\rho_{A}k_{DEP}t} \right)$$

equation (15)

The equation can be rearranged to a general form:

$$E_{t}=SSR\times\left( 1-De^{-k_{1}t}+\left( D-1 \right)e^{-k_{2}t} \right)$$

where $\mathrm{SSR}$ is the steady-state response, i.e. response as $t\to\infty$. This rearrangement involves the intermediate step:

$$E_{t}=\frac{k_{\tau}k_{R}}{k_{DEP}k_{D(obs)}}\left( 1-\frac{k_{D(obs)}\left( \rho_{A}k_{DEP}-k_{R} \right)}{k_{R}\left( \rho_{A}k_{DEP}-k_{D(obs)} \right)}e^{-\rho_{A}k_{DEP}t}+\frac{\rho_{A}k_{DEP}\left( k_{D(obs)}-k_{R} \right)}{k_{R}\left( \rho_{A}k_{DEP}-k_{D(obs)} \right)}e^{-k_{D(obs)}t} \right)$$

and the observation that the $e^{-k_{D(obs)}t}$ multiplier equals the$e^{-\rho_{A}k_{DEP}t}$ multiplier minus unity, i.e:

$$\frac{\rho_{A}k_{DEP}\left( k_{D(obs)}-k_{R} \right)}{k_{R}\left( \rho_{A}k_{DEP}-k_{D(obs)} \right)}=\frac{k_{D(obs)}\left( \rho_{A}k_{DEP}-k_{R} \right)}{k_{R}\left( \rho_{A}k_{DEP}-k_{D(obs)} \right)}-1$$

The parameters are defined as follows:

$$SSR=\frac{k_{\tau}k_{R}}{k_{DEP}k_{D(obs)}}$$

$$D=\frac{k_{D(obs)}\left( \rho_{A}k_{DEP}-k_{R} \right)}{k_{R}\left( \rho_{A}k_{DEP}-k_{D(obs)} \right)}$$

$$k_{1}=\rho_{A}k_{DEP}$$

$$k_{2}=k_{D(obs)}$$

At a saturating concentration of agonist, parameters that are agonist dependent ($D$ and $k_{1})$ are defined as follows:

$$D_{[A]\to\infty}=\frac{k_{D(obs)}\left( k_{DEP}-k_{R} \right)}{k_{R}\left( k_{DEP}-k_{D(obs)} \right)}$$

$$k_{1,[A]\to\infty}=k_{DEP}$$

The expanded equation at a saturating concentration of agonist is equation (16):

$$E_{t}=\frac{k_{\tau}k_{R}}{k_{D(obs)}k_{DEP}}\left[ 1-\frac{k_{DEP}}{k_{DEP}-k_{D(obs)}}e^{-k_{D(obs)}t}+\frac{k_{D(obs)}}{k_{DEP}-k_{D(obs)}}e^{-k_{DEP}t} \right]+\frac{k_{\tau}}{k_{DEP}-k_{D(obs)}}\left( e^{-k_{D(obs)}t}-e^{-k_{DEP}t} \right)$$

equation (16)

The initial rate of the response is $k_{\tau}$, shown by taking the limit of equation (16) as time approaches zero:

$$E_{t,\left[ A \right]\to\infty,t\to0}=k_{\tau}$$
